# Evidence for 20th century climate warming and wetland drying in the North American Prairie Pothole Region

**DOI:** 10.1002/ece3.731

**Published:** 2013-08-28

**Authors:** Brett A Werner, W Carter Johnson, Glenn R Guntenspergen

**Affiliations:** 1 Department of Environmental Studies, Public Affairs Center 309, University of IllinoisOne University Plaza, Springfield, Illinois, 62703; 2 Department of Natural Resource Management, South Dakota State UniversityNPBL 138 Box 2140B, Brookings, South Dakota, 57007; 3 U.S. Geological Survey, Patuxent Wildlife Research CenterLaurel, Maryland, 20708

**Keywords:** Climate change, cover cycle, hindcasting, North American wetlands, PPR, Prairie Pothole Region, prairie wetlands, simulation, wetlands

## Abstract

The Prairie Pothole Region (PPR) of North America is a globally important resource that provides abundant and valuable ecosystem goods and services in the form of biodiversity, groundwater recharge, water purification, flood attenuation, and water and forage for agriculture. Numerous studies have found these wetlands, which number in the millions, to be highly sensitive to climate variability**.** Here, we compare wetland conditions between two 30-year periods (1946–1975; 1976–2005) using a hindcast simulation approach to determine if recent climate warming in the region has already resulted in changes in wetland condition. Simulations using the WETLANDSCAPE model show that 20th century climate change may have been sufficient to have a significant impact on wetland cover cycling. Modeled wetlands in the PPR's western Canadian prairies show the most dramatic effects: a recent trend toward shorter hydroperiods and less dynamic vegetation cycles, which already may have reduced the productivity of hundreds of wetland**-**dependent species.

## Introduction

**Figure 1:**
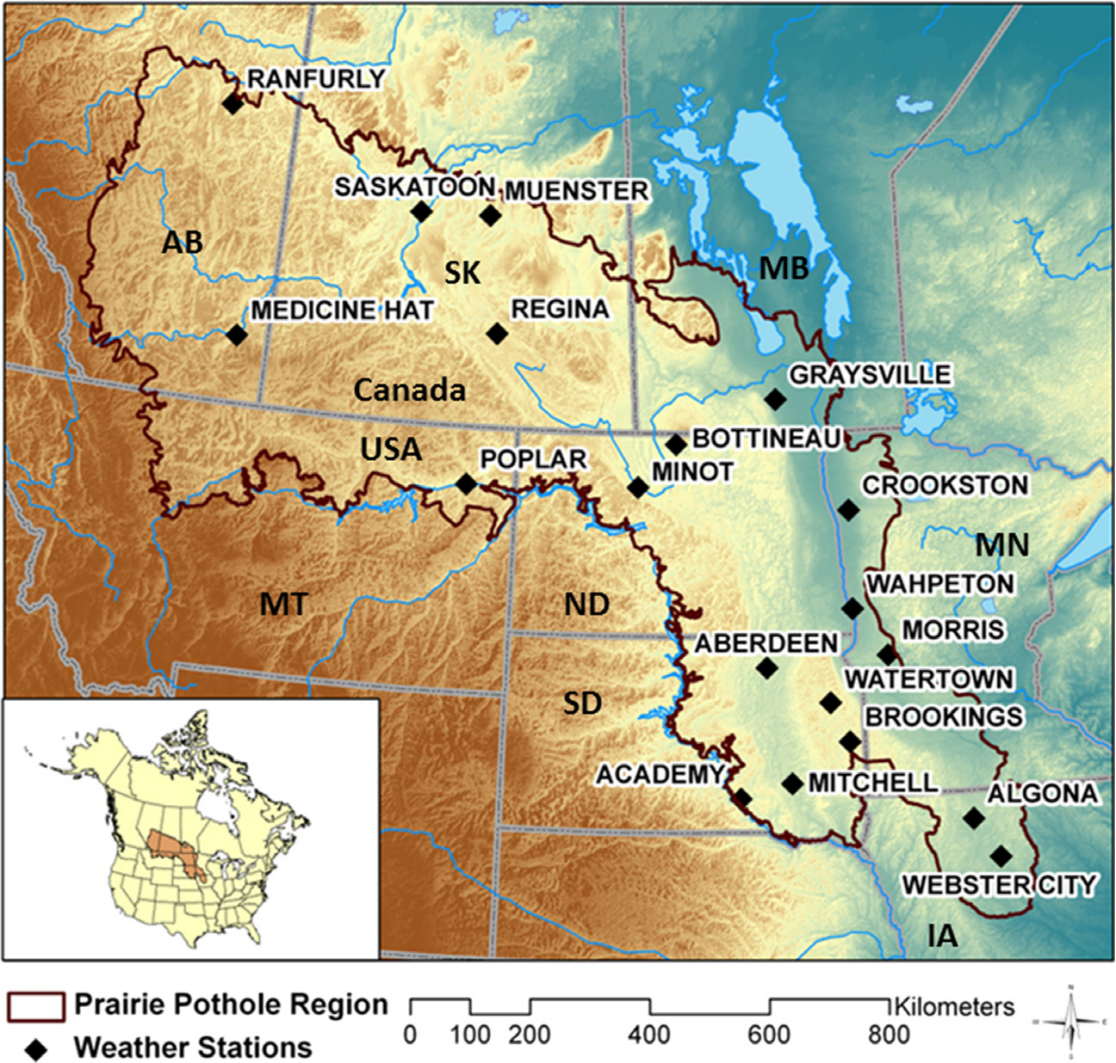
The Prairie Pothole Region (PPR) encompasses parts of five U.S. states and three Canadian provinces. Weather stations with long-term (≥100 years) climate datasets are identified (adapted from Fig. 1 in Johnson et al. 2010).

Freshwater wetlands are among the world's most valuable but vulnerable ecosystems (Costanza et al. 1997; Millenium Ecosystem Assessment 2005b; Zedler and Kercher 2005; Gitay et al. 2011). The Prairie Pothole Region (PPR) of North America, a 750,000 km^2^ area embedded with 5–8 million wetland basins of recent glacial origin occurring in a grassland climate (Fig. 1), is one of eleven large wetland ecoregions identified worldwide as conservation priorities (Keddy et al. 2009). PPR wetlands are best known as prime nesting and migratory habitat for waterfowl (Mann 1974; Murkin et al. 2000; Millenium Ecosystem Assessment 2005b; Gleason et al. 2008), but they provide other important ecosystem goods and services including flood retention, groundwater recharge, water purification, recreation, agriculture, and regional biodiversity (Galatowitsch and van der Valk 1998; van der Valk 2006; Euliss et al. 2010; Gleason et al. 2011).

Research has shown these wetlands to be especially vulnerable to climate warming (Poiani and Johnson 1991, 1993; Larson 1995; Sorenson et al. 1998; Johnson et al. 2005); projections for a doubling of atmospheric CO_2_ indicate major drying out of wetlands, thus disrupting or slowing the vegetation cover cycle that regulates wetland ecosystem primary and secondary productivity (Johnson et al. 2010). All research to date on climate change and prairie wetlands has focused on the future projections and sensitivity to climate change; here we utilize the same simulation model previously used to make future projections, but we develop a hindcast approach to detect if warming in the PPR may have been sufficient to have initiated wetland drying and thereby reduced the potential for biological productivity.

The relationship between climate's geographical and temporal variability and prairie wetland dynamics and productivity is complex. Two climatic gradients intersect to drive regional-scale biodiversity and productivity across the PPR: a steep west to east precipitation gradient and a more gradual north to south temperature gradient. These orthogonal climatic gradients produce a systematic northwest to southeast gradient of increasing wetness. Average annual precipitation and air temperature range from 400 mm and 2°C near Saskatoon, Saskatchewan to 900 mm and 9°C near Algona, Iowa (Millett et al. 2009). These climatic gradients exhibit temporal variability, with well-known wet and dry interannual cycles.

This dynamic climate, ranging from drought to deluge, interacts with variable wetland bathymetry to produce wetlands that range widely in hydroperiod and vegetation dynamics over space and time (Winter and Rosenberry, 1998; Johnson et al. 2004). Clusters of three main hydrologic permanence types (semi-permanent, seasonal, and temporary wetlands) form wetland complexes (Weller 1988; Johnson et al. 2010), which are often hydrologically connected by surface or groundwater (Winter and Rosenberry 1995; Murkin et al. 2000) and ecologically linked by highly vagile organisms seeking food, water, and cover (Naugle et al. 2001).

The prairie wetland cover cycle, described for the longer hydroperiod, semi-permanent wetlands, has been divided into four stages: dry stage with low or no standing water; regenerating stage with reflooding and vegetative propagation; degenerating stage when emergent plants begin to decline; and the lake stage with deep water and little emergent vegetation (van der Valk and Davis 1978; Johnson et al. 2004). The extremes of the cycle (drought and deluge) cause plant population turnover and nutrient release that maintain high primary and secondary productivity (van der Valk and Davis 1978; Euliss et al. 1999; Swanson et al. 2003; Johnson et al. 2005). To illustrate, low water and occasional drying of the wetland bottom during droughts stimulate plant recruitment from a diverse seed bank and mobilize nutrients through decomposition. Conversely, high water during deluges causes mortality of emergent plants and creates greater interspersion of emergent cover and open water. The transitional stages (regenerating and degenerating) are referred to as hemi-marshes covered by approximately equal proportions of open water and emergent vegetation; the hemi-marsh condition is considered to be the most productive. Total productivity during a single cover cycle may vary 20-fold (Johnson et al. 2005). Indicators of the status of the cover cycle for a given wetland and climate are the return time and the number of switches between cover cycle stages (Johnson et al. 2010). The status of the vegetation cover cycle may be the best single indicator of a wetland complex's overall (primary and secondary) productivity under a given climate (Johnson et al. 2010), and here we focus on semi-permanent wetland cover cycling to track dynamics throughout the wetland complex.

While wetlands in the PPR provide numerous ecosystem goods and services, land use change has degraded large portions of the region and made the PPR as a whole more vulnerable to future wetland losses, climate variability, and climate change. Approximately half of the natural wetland basins in the PPR were drained many decades ago for agricultural and urban uses (Tiner 1984; Dahl 2000, 2006, 2011). The proportion of wetlands drained follows the moisture (and hence cropland productivity) gradient; nearly all prairie wetlands in the subhumid climate of Iowa and western Minnesota have been drained, while at present the majority remain intact in the drier, central Dakotas and in the western Canadian Prairies (Johnson et al. 2008), although drainage has continued in both areas (Environment Canada 1991; Dahl and Watmough 2007; Bartzen et al. 2010; Oslund et al. 2010). Furthermore, the ecological functions of many remaining wetlands have been impacted by invasive species, habitat conversion, overgrazing, and by farming in dry years (Gleason and Euliss 1998; Guntenspergen et al. 2002; Gleason et al. 2003; van der Valk 2006).

The PPR has warmed in the past century at a level similar to that of the global average (Millett et al. 2009). Considerably more warming is expected in the future. The Intergovernmental Panel on Climate Change (IPCC 2007) projected a 1.8–4.0°C increase in the mean temperature of the Earth's atmosphere by the year 2100. Climate projections for the approximate PPR are temperature increases near 4.0°C and shifts of −5% to 10% in precipitation (IPCC 2007). Increased frequency of both drought and deluge are also anticipated due to an intensified hydrologic cycle (Ojima and Lackett 2002; Johnson et al. 2004). Millett et al. (2009) found that some PPR climate stations became warmer and drier during the 20th century, leading to speculation that wetlands may already exhibit a climate change signature. However, long-term monitoring data are inadequate across the PPR to detect broad trends in wetland function (Conly and Van Der Kamp 2001).

In this study, the WETLANDSCAPE (WLS) model of the wetland complex (Johnson et al. 2010) was used to simulate historic wetland conditions and to determine if recent warming has been sufficient to impact wetland function. A cover cycle index (CCI) was incorporated into WLS to evaluate a range of climate–wetland interactions (Johnson et al. 2010). Our hindcast approach compares wetland conditions between two time periods during the second half of the 20th century (1946–1975; 1976–2005).

## Methods

WLS was built on twenty years of research in wetland simulation modeling (Poiani and Johnson 1993; Poiani et al. 1995, 1996; Johnson et al. 2004, 2005; Voldseth et al. 2007, 2009; Johnson et al. 2010). WLS 1.0 uses the Stella platform (ISEE Systems, Lebanon, NH) and is a process-based, deterministic, multiple-basin wetland model constructed to address the effects of climate variability and land use on landscape-scale wetland dynamics. WLS simulates wetland surface water, groundwater, and vegetation dynamics of wetland complexes.

**Figure 2:**
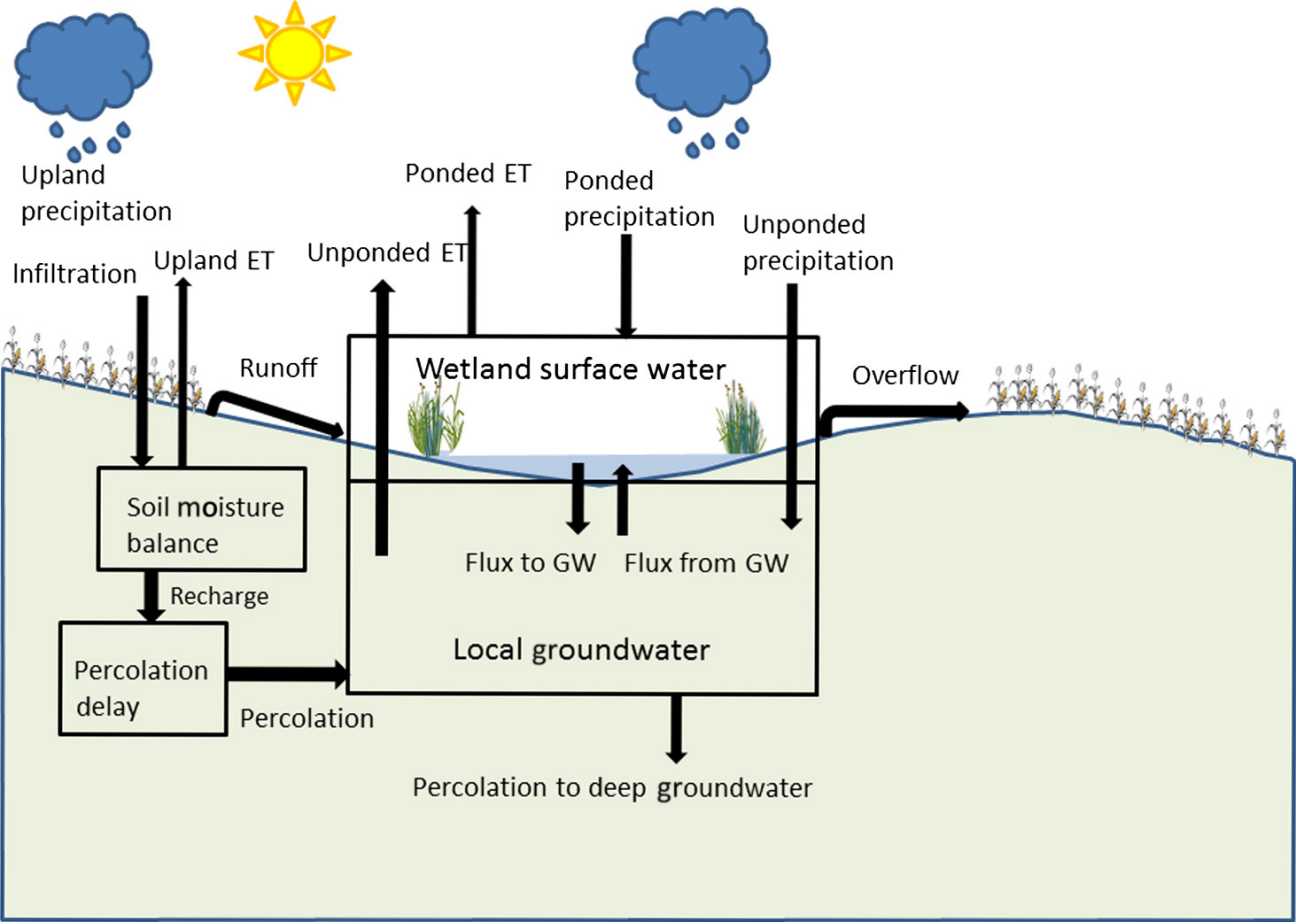
Main hydrologic variables computed by WLS for a prairie wetland complex. ET, evapotranspiration; GW, groundwater (adapted from Fig. 2 in Johnson et al. 2010).

Model inputs include wetland bathymetry, soil properties, overflow among basins, and climate (10-day averages of temperature and 10-day sums of precipitation). In WLS, water moves through the wetland complex, entering the surface water as precipitation, runoff, infiltration, percolation, and groundwater, and leaving the surface water basin through evapotranspiration, overflow, and seepage (Fig. 2). At each 10-day time step, depths of surface water and local groundwater are calculated, and from these surface water depth-duration relationships, wetland functions and processes such as the vegetation cover cycle are simulated and described.

Model calibration and validation used 20 years of observation data at the Orchid Meadows field research site, creating a modeled landscape of three temporary basins, three seasonal basins, and three semi-permanent basins (Johnson et al. 2010). Simulations of water levels and cover cycle classes for this wetland complex at each of the 19 long-term climate stations might obscure differences in soils and bathymetry (Winter 2000), but can show the relative impact of climate on wetland function (Johnson et al. 2005, 2010), particularly given that field observations occurred during both drought and deluge cycles (Johnson et al. 2004).

**Figure 3:**
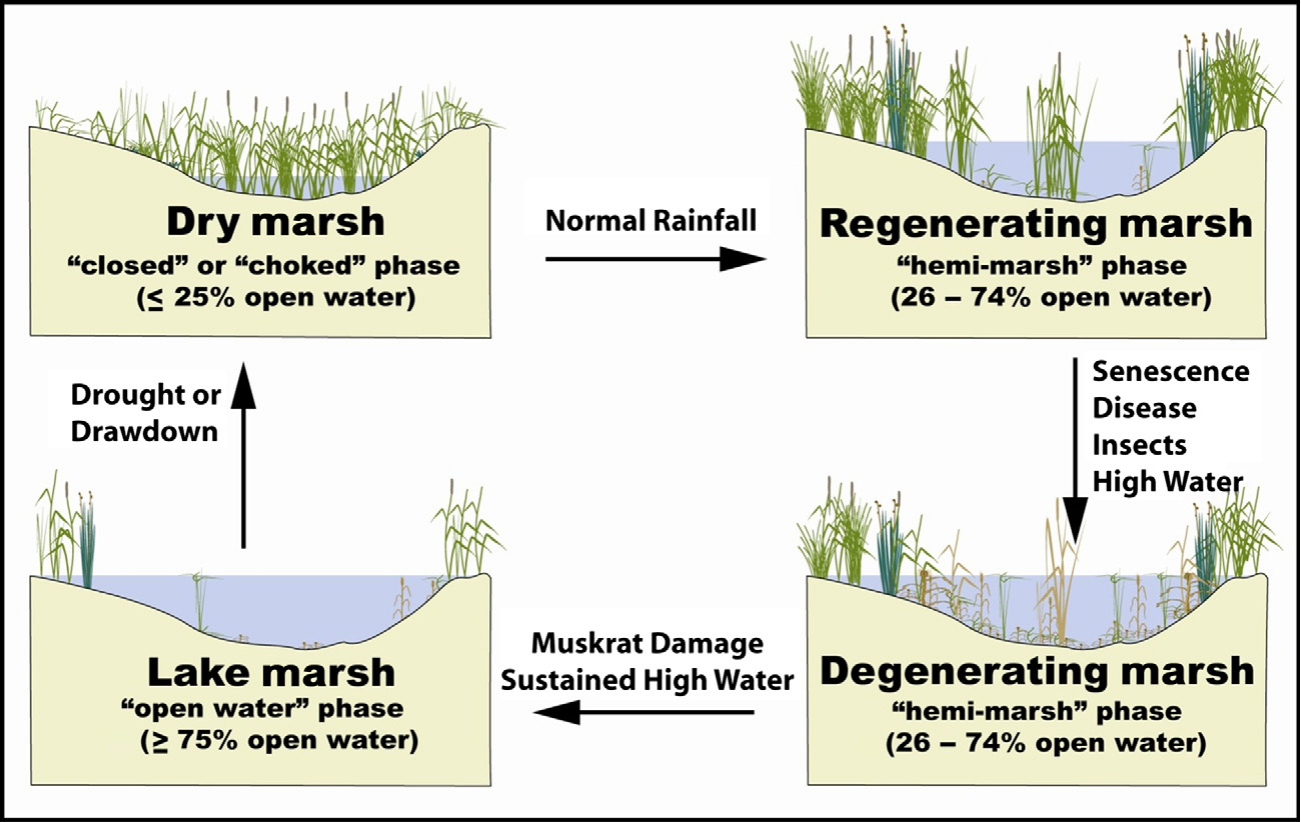
The cover cycle of semipermanent wetlands is driven by drought and deluge episodes. Extended low surface water periods create dry marsh conditions, while protracted high water floods out vegetation, leading to lake marsh conditions. Normal rainfall allows for hemi-marsh conditions, a category that includes both regenerating and degenerating marsh stages (adapted from Fig. 8 in Poiani and Johnson 1991).

In this study, WLS simulated three cover classes (lake marsh, dry marsh, and hemi-marsh combined from regenerating and degenerating stages in Fig. 3). Shifts between cover classes occur when weather conditions cause water depth and duration to cross thresholds (Table 1). Using a Markov-based approach, each of the three simulated semi-permanent basins remains in a cover class until these hydrologic conditions are met: for example, a basin in dry marsh switches to hemi-marsh when water levels are between 0.4 and 1.0 m for 1.5 years of the ice-free season, and then switches to lake marsh when the maximum depth remains above 0.75 m for 2 years. The CCI serves as the primary metric of wetland functional dynamics in WLS and is based on two variables given equal weight: the proportion of time, averaged across three semi-permanent WLS wetlands, spent in the hemi-marsh stage during the simulation period (in this case, 30 years), and the average number of cover cycle state changes (i.e., switches) between cover cycle classes over the same time period (Johnson et al. 2010).


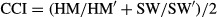
(1)

**Table 1:** Water depth thresholds required to produce switches between cover cycle stages in WLS (after Poiani et al. 1995)

Current stage	New stage	Maximum depth (meters)	Duration
Lake marsh	Hemi-marsh	<0.5	May–July
Hemi-marsh	Lake marsh	>0.75	2 years
Hemi-marsh	Dry marsh	<0.1	May–July
Dry marsh	Hemi-marsh	Between 0.4 and 1.0	1.5 years
Dry marsh	Lake marsh	>0.75	2 years

Where CCI is the Cover Cycle Index; HM is the proportion of three semi-permanent basins' timesteps spent in the hemi-marsh stage; HM' is the maximum proportion of time spent in the hemi-marsh stage across the simulations; SW is the number of switches in cover classification stage among the wetland basins; and SW' is the maximum number of switches across the simulations.

Each of the two components of CCI was scaled to a value between zero (0) and one (1) and then averaged. This yields an index that approaches one (1) when a simulated wetland complex underwent many switches among cover cycle classes and had a high proportion of timesteps in the hemi-marsh stage. The index approaches zero (0) when a wetland complex has few switches and proportionally few timesteps spent in the hemi-marsh stage. Climate drives the cover cycle (Weller and Spatcher 1965; Murkin et al. 2000), determining the speed at which semi-permanent wetlands switch among lake, dry, and hemi-marsh conditions (van der Valk and Davis 1978).

Here, we divided simulations into two 30-year periods (1946–1975 and 1976–2005). The World Meteorological Organization, recognizes 30 years as a minimum number of years to be averaged to qualify as climate data, in order to minimize the role of yearly variability. More importantly for our purposes, CCI is an output variable capable of comparing wetland functioning over decadal scales, a time scale sufficient to capture wet–dry cycles. Finally, such a split is similar to the comparisons used elsewhere to detect the onset of climate change effects on ecosystems (e.g., Cherkauer and Sinha 2010).

**Figure 4:**
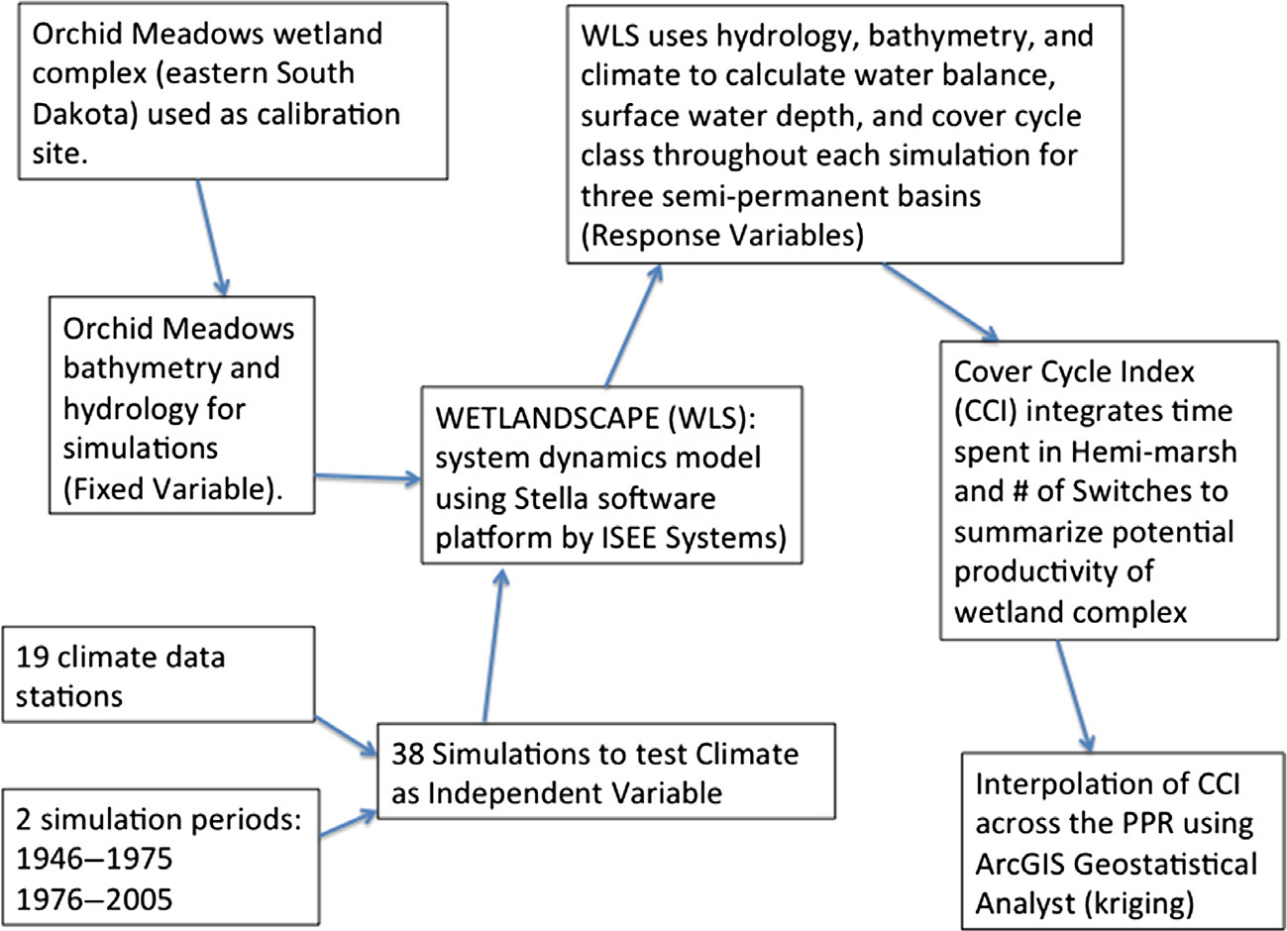
Flow diagram outlines the methodological approach, including field observations and calibration, modeling approach to hydrology and cover cycle, and simulation assumptions along with input variables.

For each of the 19 climate stations and for both of the 30-year periods, we use equation (1) to calculate CCI, and then we interpolate these CCI values across the PPR using ArcGIS 10: Geostatistical Analyst's kriging (ESRI, Redlands, CA). CCI scores are categorized as high (dark green), medium (light green), and low (yellow), corresponding to projected productivity of the semi-permanent wetlands, based on the cover cycle. Color coding is based only on the gradation of the CCI, not on other measures (i.e., time spent in dry marsh or lake marsh). To interpret patterns in clusters of climate stations, we grouped the climate stations by subregion: northwest (Poplar, MT; Medicine Hat, AB; Saskatoon, SK; Regina, SK; Ranfurly, AB; Muenster, SK), south central (Aberdeen, SD; Academy, SD; Mitchell, SD; Watertown, SD; Brookings, SD), northeast (Bottineau, ND; Minot, ND; Graysville, MB; Wahpeton, ND; Crookston, MN), and southeast (Morris, MN; Webster City, IA; Algona, IA). Figure 4 summarizes the methodological approach in a flow diagram.

## Results

**Figure 5:**
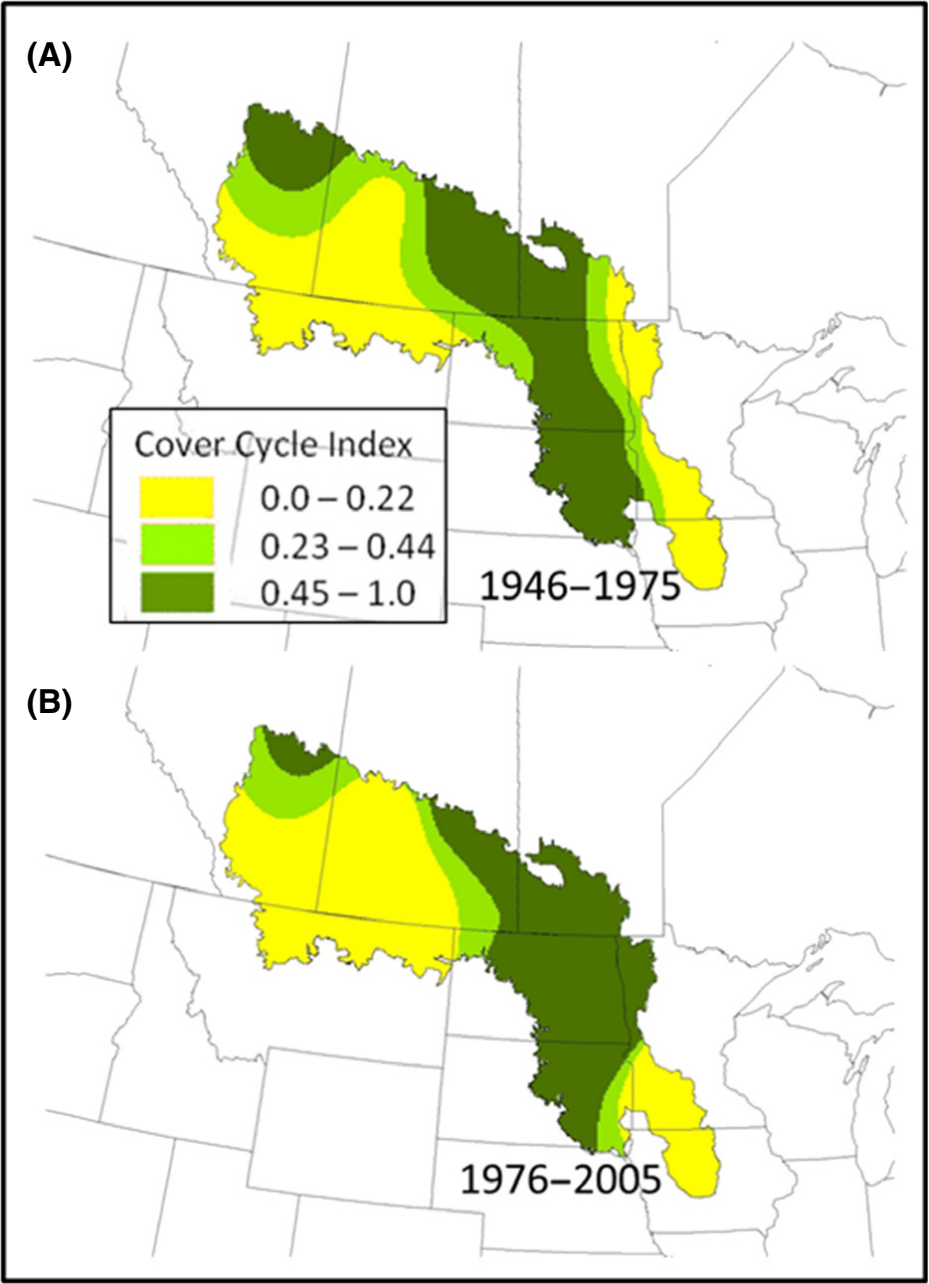
Broad patterns of cover cycle dynamics across the PPR during two 30-year periods based on the CCI. (A) shows the 1946–1975 simulation period. (B) shows the 1976–2005 simulation period.

The hindcast analysis detected two main geographic shifts in CCI scores across the PPR during the second half of the 20th century. First, the climate of the second period in the western Canadian prairies was sufficiently drier and warmer to enlarge from 40% (1946–1975) to 47% (1976–2005) the proportional area of the PPR covered by the least productive CCI category, shown in yellow (Fig. 5). The area of the intermediate CCI category (light green) decreased accordingly from 21% to 13%. Second, the most productive area of the PPR, shown as a dark green swath in the eastern Dakotas and southeastern Saskatchewan (Fig. 5A), did not change in areal extent but shifted eastward into northwestern Minnesota and westward in central North Dakota (Fig. 5B). Areas bounding this swath had lower CCI scores because the climate was suboptimal for wetland productivity: too dry in the west and too wet in the east. In general, precipitation decreased for most of the northwestern stations between the two periods, but increased for most of the other stations (Table 2). Air temperatures increased for the northwestern stations, but changed little for most of the other stations.

**Table 2:** Climate data comparisons for 19 PPR weather stations that show the 30-year means for yearly precipitation (mm) and 30-year means for minimum and maximum daily air temperatures (^°^C)

Station	Prec 46-75	Prec 76-05	Tmin 46-75	Tmin 76-05	Tmax 46-75	Tmax 76-05
Poplar, MT	406.2	402.2	−2.03	−1.03	13.03	14.23
Medicine Hat, AB	417.6	402.3	−1.47	−0.70	11.49	12.47
Saskatoon, SK	435.6	411.2	−4.59	−3.62	7.46	8.54
Regina, SK	467.6	450.3	−4.29	−3.34	8.23	9.23
Ranfurly, AB	518.1	516.7	−4.37	−3.06	7.13	8.24
Muenster, SK	465.2	477.7	−4.81	−3.63	6.06	7.07
Aberdeen, SD	540.7	620.5	−0.79	0.19	12.71	12.83
Academy, SD	640.5	696.3	1.74	1.57	15.97	15.60
Mitchell, SD	642.1	711.2	1.68	1.94	15.23	14.51
Watertown, SD	660.3	668.0	−0.62	0.32	11.73	12.21
Brookings, SD	641.3	706.7	−0.57	−0.10	12.71	12.07
Bottineau, ND	533.0	526.3	−3.81	−2.75	9.15	9.75
Minot, ND	640.5	696.3	−2.28	−1.43	10.16	10.62
Graysville, MB	614.0	650.0	−3.67	−2.68	8.36	9.33
Wahpeton, ND	653.3	669.3	−0.31	0.26	12.07	12.11
Crookston, MN	540.7	620.5	−1.94	−1.44	10.48	10.37
Morris, MN	713.5	774.2	−0.39	−0.12	11.14	11.50
Webster City, IA	891.7	1022.3	2.50	2.53	14.53	14.67
Algona, IA	870.8	927.0	1.95	2.22	13.92	13.61

Climate variable abbreviations are Prec, precipitation; Tmin, minimum temperature; Tmax, maximum temperature; 46-75, 1946–1975; 76-05, 1976–2005. State and province abbreviations are IA, Iowa; MN, Minnesota; MT, Montana; ND, North Dakota; SD, South Dakota; AB, Alberta; MB, Manitoba; SK, Saskatchewan.

**Figure 6:**
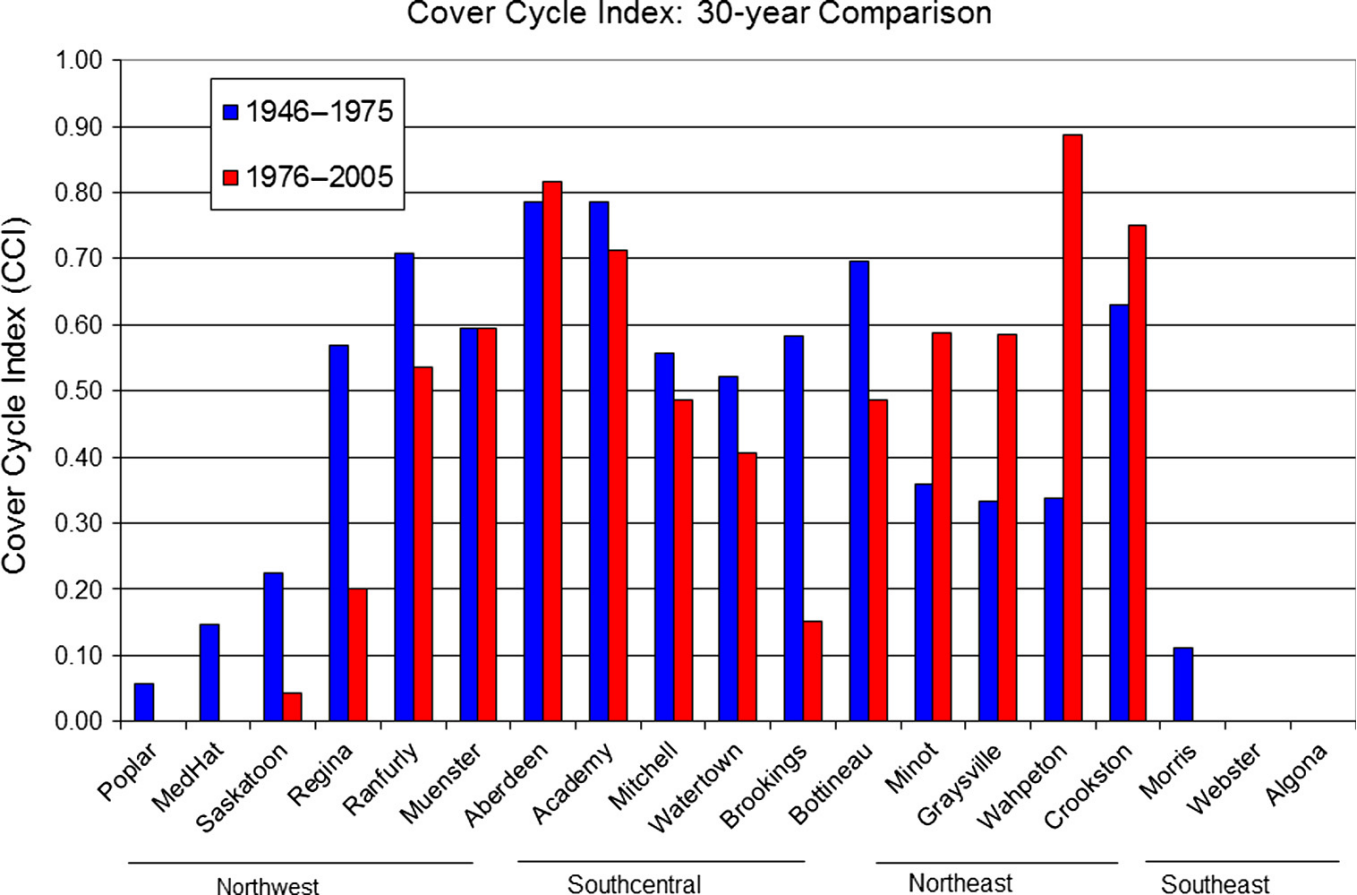
omparison of CCI scores between two 30-year periods for 19 weather stations grouped by subregion in the PPR.

The overall effect of the climate differential between periods was to shift the most productive conditions eastward and to lower potential productivity in the west. The CCI remained unchanged at three, increased at five, and decreased at 11 of the 19 weather stations used in the analysis (Fig. 6, Table 3) indicating a widespread decline in climate favorability in the latter period.

**Table 3:** Cover cycle index (CCI) values are shown for each PPR weather station and period, along with component variables

Station	1946–1975					1976–2005				
	
	Lake	Hemi	Dry	Switches/basin	CCI	Lake	Hemi	Dry	Switches/basin	CCI
Poplar, MT	0	1	99	0.67	0.06	0	0	100	0.00	0.00
Medicine Hat, AB	0	10	90	0.67	0.15	0	0	100	0.00	0.00
Saskatoon, SK	2	6	92	2.33	0.22	0	2	98	0.33	0.04
Regina, SK	15	22	64	5.00	0.57	2	10	87	1.33	0.20
Ranfurly, AB	25	41	34	4.33	0.71	31	30	39	3.33	0.53
Muenster, SK	20	34	46	3.33	0.57	9	41	50	2.33	0.57
Aberdeen, SD	21	51	28	4.00	0.78	39	56	4	3.67	0.82
Academy, SD	32	34	34	6.33	0.79	58	31	11	5.67	0.71
Mitchell, SD	13	25	62	4.33	0.56	66	13	21	5.00	0.49
Watertown, SD	64	36	0	2.33	0.52	81	17	2	3.33	0.41
Brookings, SD	54	30	16	4.00	0.58	92	8	0	1.00	0.15
Bottineau, ND	14	39	46	4.33	0.70	19	23	58	3.67	0.49
Minot, ND	28	15	57	3.00	0.36	42	31	28	4.00	0.59
Graysville, MB	59	14	26	2.67	0.33	60	40	0	2.67	0.59
Wahpeton, ND	57	13	31	3.00	0.34	31	61	8	4.00	0.89
Crookston, MN	48	52	0	1.67	0.63	36	54	10	3.00	0.75
Morris, MN	93	7	0	0.67	0.11	100	0	0	0.00	0.00
Webster City, IA	100	0	0	0.00	0.00	100	0	0	0.00	0.00
Algona, IA	100	0	0	0.00	0.00	100	0	0	0.00	0.00

Variables include the percentage of time spent in each of the three cover cycle classes (Lake: lake marsh; Hemi: hemi-marsh; Dry: dry marsh), and the average number of switches per semipermanent basin (an average of three modeled basins). The time spent in hemi-marsh and the number of switches are the two variables used to calculate CCI, and the time spent in lake marsh and dry marsh enable comparisons of the relative wetness or dryness of model wetlands over each 30-year simulation period. See Table 2 for state and province abbreviations.

Weather stations were grouped by subregion to summarize CCI patterns in response to climate during the two periods. The northwest subregion exhibited the most striking and consistent change; CCI decreased at five of the six stations. The CCI at the Regina, Saskatchewan station, centrally located in the northwest, decreased the most, from 0.57 to 0.20 (Fig. 6). This station experienced effective drying between the two simulation periods, shifting from a Lake-Hemi-Dry Marsh ratio of 15:22:64 to one of 2:10:87, and from an average of 5.0 modeled cover cycle class switches per basin to 1.3 (Table 3). The rather dramatic change in CCI for Regina and several other prairie Canada stations was caused by rather small changes in temperature and precipitation. For example, mean precipitation at Regina decreased very little between the periods, from 468 to 450 mm (Table 2). Similarly, average daily minimum and maximum temperatures as Regina warmed from −4.3 to −3.3°C and from 8.2 to 9.2°C, respectively (Table 2).

The results at other northwestern stations such as Saskatoon, Saskatchewan (0.22 to 0.04) and Poplar, Montana (0.06 to 0.00) reflect significant differences in the relative CCI values (Table 3), but are smaller absolute changes in CCI than at Regina because the CCI values of these climatically drier stations in the 1945–1975 period were near the lower end (0.0) of the CCI scale. These stations remained primarily in the dry marsh stage during the 1976–2005 period. Again, WLS simulations suggest that climatic differences between the earlier and later simulation periods have likely affected wetland function, in this case, decreasing the likelihood of the subregion's wetlands cover cycling and spending time in hemi-marsh.

CCI scores also declined sharply for weather stations in the southcentral subregion, but for a different reason than the declines in the northwest. For example, CCI at the Watertown and Brookings stations in South Dakota declined between the periods from 0.52 to 0.41 and from 0.58 to 0.15, respectively (Fig. 6). Stations in this subregion are relatively wet but became wetter in the second simulation period (Table 2). Both stations spent proportionally more time in the lake marsh stage (Table 3). Brookings never switched to the dry marsh stage in the second period. Climate changes that produced these shifts were precipitation based more than temperature based (Table 2). These results, along with the continued effective wetness in southwestern Minnesota and Iowa, prevented the eastward shifting of optimal (dark green) CCI values to counterbalance the effective drying in the northwestern subregion.

Changes in the balance between precipitation and temperature at some northeast stations, especially at Minot (ND), Graysville (MB), and Wahpeton (ND), produced markedly higher CCI scores (Fig. 6) because conditions either became slightly wetter or slightly drier (Table 2). Only the northeastern subregion improved in cover cycle dynamics in the 1976-2005 period. These results offer a counterpoint to the apparent decline of potential productivity in the northwest.

The stations in the southeast subregion, the wettest of all subregions, remained characteristically unproductive throughout the 60-year simulation period by remaining in the lake marsh stage and rarely able to cycle through the dry and hemi-marsh cover classes (Fig. 6). CCI scores for the Webster and Algona stations never rose above zero in either period (Table 3). Air temperature differed little at these stations between the periods; however, both stations were considerably wetter in the later period (Table 2).

In summary, the temperature and precipitation changes between the two, 30-year normal periods varied considerably across the relatively small PPR. Interestingly, the climatically drier weather stations became drier and warmer, while the wetter regions became wetter with minimal change in temperature. The drier conditions in the northwest and wetter conditions in the southeast combined to steepen the west to east climatic gradient caused by the rain shadow of the Rocky Mountains. The climatic differences between periods produced relatively large changes in CCI, some stations cycling slower and some cycling faster.

## Discussion

Our hindcast modeling indicates that the recorded climate shifts during the two periods was of sufficient magnitude to have produced large changes in wetland productivity, as measured by the CCI index, in some subregions of the PPR. The analysis presents a complex, yet patterned, message regarding how semi-permanent wetlands have likely responded to recent shifts in climate. The clearest signal came from the Canadian prairies and adjacent U.S. border areas identified during earlier analyses as highly sensitive to climate change (Johnson et al. 2005, 2010). An important secondary signal came from the historically wetter northeastern PPR climate that became more dynamic in the second time period.

The trends in the northwest subregion of the PPR deserve special attention for three reasons: globally, ecosystems at higher latitudes are expected to warm more than those at lower latitudes (IPCC 2007); the climatically dry western Canadian prairies historically have been characterized as a “boom or bust” breeding ground for waterfowl, situated on a climatic “knife edge” highly sensitive to warming and drying (Johnson et al. 2010); analysis of climate data show this PPR subregion to have warmed the most during the 20th century (Millett et al. 2009). Reductions in productivity simulated by WLS associated with a warmer and drier climate in the northwest subregion during the period 1976–2005 provide evidence that the “knife edge” threshold may have been crossed recently, meaning that these areas may already be too dry for cover cycling in the context of a 30-year simulation period.

Has the more favorable climate for wetlands in the northeastern PPR counterbalanced the simulated decline in the northwestern subregion? If functional wetlands were abundant in the northeast the answer could be “yes.” However, wetland inventory data show that the western PPR has the highest functional (undrained) wetland densities and grassland habitat important for vertebrate life cycles, while in the east nearly all wetlands have been drained (Dahl 2000, 2006, 2011) and associated grassland plowed up for agriculture (Samson et al. 2004; Hoekstra et al. 2005; Stephens et al. 2005). So gains in the east could only compensate for biological productivity declines in the west if large numbers of drained wetlands were restored and watersheds replanted with grassland (Galatowitsch and van der Valk 1998; Zedler 2003). Until and even after such a trend toward restoration is realized, grassland and wetland conservation in the Dakotas is important (Loesch et al. 2012). Additionally, Wright and Wimberly (2013) indicates that land use conversion of grasslands and wetlands to corn–soybean agriculture in the eastern Dakotas is increasing, further compounding the effects of climate change on prairie wetland ecosystems.

The pattern of change in CCI between the two 30-year periods bears a striking resemblance to WLS projections comparing CCI based on a 100-year (20th century) climate data set for the PPR and that projected by a 2°C increase in air temperature applied uniformly across all weather stations (Johnson et al. 2010). The area of the lowest CCI scores expanded in the western Canadian prairies and the most favorable category moved eastward. Splitting of the data set into two 30-year periods revealed that the magnitude of climate change late in the 20th century was sufficient to cause a productivity drop comparable to that projected several decades into the future. In short, conditions simulated for the warmer future may have already arrived in the northernmost portion of the PPR.

The loss of wetland function is likely to have the largest effect on animals requiring long wetland hydroperiods (waterfowl, amphibians, and shorebirds) compared to shorter hydroperiods (invertebrates, Johnson et al. 2010). These findings align with other research done on Canada's western prairie provinces (Clair 1998; van Kooten et al. 2011; Withey and van Kooten 2011) where climate warming and human modifications have reduced the flows of major rivers during the summer months (Schindler and Donahue 2006). Dry regions, such as Canada's western prairie provinces, were identified in the Millenium Ecosystem Assessment as hotspots for future environmental degradation because of the effects of climate warming and human activity (Millenium Ecosystem Assessment 2005a).

The global importance of wetlands in general, and Prairie Pothole wetlands in particular, cannot be overstated. The biodiversity that PPR wetlands support, regionally for resident species, and globally for migratory bird species, are the primary reason to closely monitor the status, trends, and outlook of climate impacts on wetland functioning. Given that these wetlands are already degraded in certain geographic sectors of the PPR, and under continual threat to be further degraded in the remaining sectors, the hindcast modeling approach of this paper is a valuable tool to discern and interpret the recent impacts of climate change on wetlands, especially in the absence of long-term, region-wide field data. Hindcast simulation modeling should be a key addition to the wetland ecologist's toolbox for determining the vulnerability of global wetlands to climate change because of the geographic, temporal, and financial limitations on field research.

We recommend that future research combine modeling and monitoring in tandem to develop an “early warning” detection system for climate change effects on prairie wetlands. Current wetland monitoring across the PPR is inadequate to verify these WLS hindcast simulations. Only three long-term wetland monitoring field sites (St. Denis, SK; Cottonwood Lake, ND; Orchid Meadows, SD) with greater than 10 years of continuous records exist in the PPR; records from none of these three stations spans the 60-year period examined in this study. And these sites represent an ad hoc network and are not part of a institutionalized monitoring effort. Further, data from the 4-mile^2^ (Johnson and Grier 1988) and May Pond data sets (Cowardin et al. 1995), while spatially robust and containing many years of data, offer only a snapshot of wetland conditions in any year since water condition in these wetlands are only monitored once a year, and are thereby less useful to judge the impacts of climate warming on wetland water budgets in ways that could make spring ice-out times earlier and stronger summer drawdowns due to increased evapotranspiration. Wetland scientists have called for more extensive long-term monitoring across the PPR to detect future climate change impacts (Conly and Van Der Kamp 2001; Johnson et al. 2004, 2005; Dahl and Watmough 2007; Johnson et al. 2010; Niemuth et al. 2010). Monitoring data from these long-term stations and other field monitoring programs need to be institutionalized, standardized where possible, and made available to the wetland science community. Bringing selected wildlife refuges in North America that possess historic wetland data into an integrated monitoring system would be one approach to begin to fill the large geographic gaps in the current network.

Modeling, as demonstrated in this paper, can identify alternate futures based on the best science and data available, separate the climate signal from the considerable “noise” present in the human-impacted prairie wetland system, and make monitoring more streamlined and purposeful by suggesting where and what to monitor. Conversely, monitoring is needed to build accurate models, check projections, and to determine when models have attained adequate levels of predictability to justify scaling back or eliminating monitoring to reduce costs or redirect effort to management solutions. Thus, monitoring and modeling should be adapted as an iterative process that improves model forecasting and optimizes the cost of extensive and long-term monitoring programs. Hindcast modeling and recent analysis of climate data provide strong arguments for immediate development of an early warning system to detect and understand the nature of threats to this valued international resource.

## Conclusions

Modeling indicates that freshwater wetlands of North America's PPR may have already been impacted by climate change during the past 3 decades. This research has shown that 20th century warming in the northern latitudes of the PPR identified by previous studies has been sufficient to have had significant impact on wetland cover cycling, a key indicator of wetland productivity and biodiversity. Because of climate change, the northwestern PPR's wetlands of Canada's Western Prairie Provinces may have been contributing less to migratory populations of avifauna than it has in the past. An expanded program of modeling and monitoring in tandem is proposed for the PPR to produce an early warning system needed to check on model predictions and to streamline monitoring.
